# Insights Into the Interplay Between the Senescent Cells and Immune Cells

**DOI:** 10.1111/acel.70698

**Published:** 2026-09-03

**Authors:** Martin Jaros, Marco Schmidt, Lavinia Neubert, Jan‐Christopher Kamp, Sibylle von Vietinghoff, Jan Hinrich Bräsen, Roland Schmitt, Anette Melk

**Affiliations:** ^1^ Department of Pediatric Kidney, Liver, Metabolic Diseases and Neuropediatrics Hannover Medical School Germany; ^2^ Hannover Biomedical Research School Hannover Medical School Hannover Germany; ^3^ Interdisciplinary Transplantation, Children's Hospital Hannover Medical School Hannover Germany; ^4^ Institute for Pathology, Hannover Medical School Hannover Germany; ^5^ Biomedical Research in Endstage and Obstructive Lung Disease Hannover German Center for Lung Research Hannover Germany; ^6^ Department of Respiratory Medicine Hannover Medical School Hannover Germany; ^7^ Nephrology Section, Medical Clinic 1 University Hospital Bonn Bonn Germany; ^8^ Nephropathology Unit, Institute for Pathology Hannover Medical School Hannover Germany; ^9^ Department of Nephrology Hannover Medical School Hannover Germany; ^10^ Klinik für Innere Medizin IV mit den Schwerpunkten Nieren‐ Und Hochdruckkrankheiten University Medical Center Schleswig‐Holstein Kiel Germany

**Keywords:** cellular senescence, immune cells, inflammaging, kidney aging, macrophages, senolysis

## Abstract

Aging kidneys exhibit accumulation of senescent cells together with sterile low‐grade inflammation. However, the spatial organization of senescence‐associated immune cell accumulation in the aging kidney remains poorly defined. We systematically analyzed kidneys from young, middle‐aged, and aged mice, focusing on the spatial relationship between senescent tubular cells and distinct immune cell populations. Senescent tubular cells showed significant local enrichment of immune cells, with macrophages representing the most prominent associated immune cell population. This spatial association was more closely linked to p16^*Ink4a*^ burden as an indicator of biological aging than to chronological age alone, as kidneys with higher p16^*Ink4a*^ levels displayed enhanced inflammatory and SASP‐associated transcriptional signatures. Complementary spatial transcriptomic analyses identified a cortex‐restricted senescence‐associated neighborhood enriched for inflammatory, macrophage‐related, and failed‐repair tubular transcriptional programs, supporting the presence of localized senescence‐associated inflammatory niches at the transcriptomic level. Both, genetic and pharmacological senolytic interventions reduced senescent‐cell burden and decreased immune cell accumulation in aged kidneys. However, macrophages remained preferentially localized near residual senescent tubular structures after senolysis, consistent with persistent local immune senescent cell interactions. Our findings provide quantitative spatial evidence that senescence‐associated inflammatory niches emerge in the aging kidney cortex and can be modulated by senolytic intervention. Together, these results establish a spatial framework for renal inflammaging and highlight senescence‐associated inflammatory microenvironments as potential therapeutic targets in kidney aging.

## Graphical abstract

Kidney aging is associated with increased proximal tubular senescence and leukocyte infiltration. Senolytic treatment reduces senescent cell burden and overall immune cell infiltration. However, macrophages remain enriched around residual senescent cells.

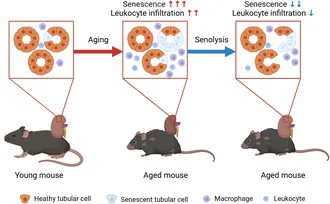

## 1 Introduction

As the global population ages, understanding the mechanisms underlying age‐related kidney decline is becoming increasingly relevant. Even in the absence of overt disease, aged kidneys exhibit structural alterations such as nephron loss, interstitial fibrosis, tubular atrophy, and glomerulosclerosis (Hommos et al. 2017). These changes are often accompanied by immune cell infiltration, particularly in areas with tubular atrophy and interstitial fibrosis (Yadav et al. 2023). This sterile, low‐grade, chronic inflammation in the absence of infection, known as *inflammaging* is a hallmark of aging tissues (Sinning et al. 2023). Alongside these structural changes, kidney function steadily declines with age (Waas et al. 2021). The diminished regenerative capacity of the aged kidney predisposes older individuals to acute injury and chronic kidney disease (CKD) (Schmitt and Cantley 2008), a phenomenon linked to the accumulation of senescent cells (Braun et al. 2012; Melk et al. 2004; Rex et al. 2023; Schmitt and Melk 2017).

Cellular senescence is a state of stable cell cycle arrest triggered by replicative exhaustion or stressors such as DNA damage and oxidative stress. Because no single marker reliably identifies senescent cells across all contexts, composite approaches are often required (Kudlova et al. 2022). In the kidney, the cyclin‐dependent kinase inhibitor p16^*Ink4a*^ has been shown to be a reliable and widely used marker, frequently upregulated in both aging and disease (Melk et al. 2005, 2004; Westhoff et al. 2008). Ablation of p16^*Ink4a*^‐positive cells improves repair after ischemic injury and extends graft survival (Braun et al. 2012), highlighting a causal role in functional decline. Senescent cells remain metabolically active, gain apoptosis resistance, and acquire a senescence‐associated secretory phenotype (SASP) composed of cytokines, chemokines, growth factors, and matrix‐remodeling enzymes (López‐Otín et al. 2013). SASP factors promote inflammation, fibrosis, and impaired repair (Coppé et al. 2010). The interaction between senescent cells and immune cells in the aging kidney, therefore, likely forms a self‐perpetuating circle where senescent cells, in particular tubular cells, become an important mediator of inflammation leading to recruitment and activation of immune cells through the SASP (Sinning et al. 2023). In turn, the recruited immune cells promote further senescence through inflammatory mediators and oxidative stress (Hernandez‐Segura et al. 2018).

Senolysis, the targeted elimination of senescent cells, has emerged as a promising therapeutic strategy to restore tissue regeneration and function in aging organs. Senolytic agents, such as the BCL‐2/BCL‐xL inhibitor ABT‐737, reduce senescent cell burden in aged murine kidneys and enhance regeneration while limiting fibrosis (Mylonas et al. 2021; Y. Zhu et al. 2015). Genetic models such as the INK‐ATTAC mice allow inducible, p16^*Ink4a*^‐specific cell ablation and improve multiple age‐related phenotypes, including attenuation of glomerulosclerosis (Baker et al. 2011). However, despite evidence that senescent cells and immune cells interact in the aged kidney, it remains unknown how senolytic interventions alter the arrangement between these cell types.

In this study, we investigate the spatial relationship between senescent tubular cells and distinct immune cell populations in the aging kidney and assess how senolytic interventions reshape this microenvironment. By integrating quantitative histology, RNA in situ hybridization (RNA‐ISH), NanoString‐based transcriptomics, and supplementary Visium spatial transcriptomics, we aim to define whether senescence‐associated inflammatory changes in the aging kidney are spatially organized into localized inflammatory niches. In addition, we explore whether interindividual differences in senescence burden are associated with distinct inflammatory and immune cell signatures independent of chronological age.

## 2 Methods

### 2.1 Experimental Animals

We used a total of 34 old wild‐type male mice (22–24 months) and 10 young wild‐type male mice (3–4 months) of the C57BL/6J strain. In addition, two young wild‐type female C57BL/6 mice (3–4 months) were included exclusively in the NanoString dataset.

Additionally, we used mice from an INK‐ATTAC colony (breeding pairs of INK‐ATTAC mice were generously provided by Dr. Jan van Deursen through Unity Biotechnology). For the pharmacological senolysis model, we treated additional aged C57BL/6 mice (20–22 months) from the INK‐ATTAC colony (none of which had previously received AP20187) with intraperitoneal injections of BCL‐2/BCL‐xL inhibitor ABT‐737 (ApexBio, Houston, USA) or vehicle. The first dose was administered 42 days prior to tissue collection, followed by two additional doses at 14‐day intervals. The final dose was given 2 weeks before harvest. Each group comprised five mice.

For the genetic senolysis model based on (Baker et al. 2011), we used additional middle‐aged transgenic INK‐ATTAC mice (15–16 months) on a C57BL/6 background. Animals received five intraperitoneal injections of either the compound AP20187 (to induce apoptosis in p16^*Ink4a*^ expressing cells) or vehicle, administered over a period of 1 month on Days 3, 7, 10, 14, and 27. Tissues were collected on Day 30. Each group comprised five mice.

Mice deficient in p16^*Ink4a*^ expression (20–22 m old, p16^*Ink4a−/−*^) on a 129/Ola‐C57BL/6J background were used as a genetic model of p16^*Ink4a*^ deficiency in this study. These mice were originally described by Krimpenfort et al. (2001) and contain an allele resulting in loss of p16^*Ink4a*^ expression. A total of five p16^*Ink4a−/−*^ mice and five wild‐type (WT, p16^*Ink4a+/+*^) littermates from the same colony were analyzed.

Kidneys were harvested without perfusion and processed identically across all experimental groups. For formalin‐fixed paraffin‐embedded (FFPE) samples, tissues were fixed in 4% formalin for 24 h, dehydrated using a graded ethanol and xylene series, and embedded in paraffin. For cryopreserved samples, tissues were equilibrated in 15% and 30% sucrose, embedded in OCT, and stored at −80°C. Analyses in this study were restricted to the kidney cortex, based on its known susceptibility to age‐associated and senescence‐related changes (Melk et al. 2005; Melk et al. 2004; X. L. Wang et al. 2014).

### 2.2 SA‐β‐Gal Staining

Cryosections were fixed in a freshly prepared solution of 2% formaldehyde and 0.2% glutaraldehyde in PBS at room temperature for 10 min. After rinsing with PBS, sections were incubated at 37°C in a humidified chamber (without CO_2_) with freshly prepared SA‐β‐gal staining solution (pH 6.0) for 5 h. The staining solution consisted of 40 mM citric acid/sodium phosphate buffer, 5 mM potassium ferrocyanide, 5 mM potassium ferricyanide, 150 mM sodium chloride, 2 mM magnesium chloride, and 1 mg/mL 5‐bromo‐4‐chloro‐3‐indolyl β‐D‐galactopyranoside (X‐gal; stock solution prepared in dimethylformamide).

### 2.3 Immunohistochemistry

Paraffin‐embedded kidney sections were first deparaffinized, followed by antigen retrieval in 10 mM sodium citrate buffer (pH 6.0) at 95°C for 20 min. Blocking was then performed with 5% BSA. Sections were incubated overnight at 4°C with the primary antibody. On the following day, staining was visualized using Alexa Fluor 488‐ or 555‐conjugated secondary antibodies, with DAPI included in the mounting medium.

Quantification of positive cells was performed in 10 nonoverlapping fields from the kidney cortex at 200× magnification. Data were expressed as either the number of positive cells or the fraction of positive area. Cell quantification was calculated as the percentage of positive cells relative to the total number of cells per field. The list of antibodies used is provided in Table 1.

**TABLE 1:** Primary and secondary antibodies used for immunohistochemistry.

Target	Host species	Type	Dilution	Supplier	Catalog number
CD45	Rat	Primary	1:100	BD Pharmingen	5,550,539
F4/80	Rabbit	Primary	1:100	Cell Signaling	70076S
CD3	Rabbit	Primary	1:100	Dako	A0452
CD138	Rabbit	Primary	1:100	Proteintech	10,593–1‐AP
CD86	Rat	Primary	1:100	Cell Signaling	19589S
CD206	Goat	Primary	1:100	Thermo Fischer	PA5‐46994
Kim1	Goat	Primary	1:100	Biotechne	AF1817
NKp46/NCR1	Goat	Primary	1:100	Biotechne	AF2225
Alexa 555	Goat anti‐rat	Secondary	1:250	Invitrogen	A21434
Alexa 555	Goat anti‐rabbit	Secondary	1:250	Invitrogen	A21428
Alexa 555	Donkey anti goat	Secondary	1:250	Invitrogen	A21432
Alexa 488	Donkey anti‐rabbit	Secondary	1:250	Invitrogen	A21206
Alexa 488	Goat anti‐rat	Secondary	1:250	Invitrogen	A11006

### 2.4 RNA In Situ Hybridisation

Detection of p16^*Ink4a*^ mRNA was performed using the RNAscope 2.5 HD detection kit together with the RNAscope Probe‐Mm‐Cdkn2a (accession number NM_009877.2), both from Advanced Cell Diagnostics (Newark, USA). RNA in situ hybridization (RNA‐ISH) was conducted on FFPE sections according to the manufacturer's protocol. Briefly, sections were deparaffinized, subjected to heat‐induced epitope retrieval, and then treated with protease prior to probe hybridization. The signal was visualized using the Fast Red chromogen. Quantification of positive areas was performed on five nonoverlapping images per sample at 200× magnification, using image thresholding to determine the percentage of positive pixels. The specificity of this method was confirmed by the absence of a signal in p16^*Ink4a−/−*^ mice (Figure S1).

### 2.5 Picrosirius Red

Paraffin sections were deparaffinized and rehydrated in tap water for 10 min. Background staining was reduced by incubating sections in 0.2% phosphomolybdic acid for 5 min. Slides were then stained with 0.1% Sirius Red in saturated picric acid for 2 h at room temperature. After a brief rinse in 0.01 N HCl and subsequent washing in water until the excess dye was removed, slides were mounted in water and imaged immediately. Brightfield images were acquired at 200× magnification, with 10 nonoverlapping fields per sample.

### 2.6 Spatial Analysis of Senescent Cells and Different Immune Cell Populations

To assess the spatial relationship between senescent cells and immune infiltration, we used a grid‐based quantification approach based on (Walschus et al. 2011) and adapted it to our imaging format and marker combinations. Manual cell counts were performed within a standardized grid using ImageJ (NIH). Brightfield images of SA‐β‐gal staining were merged with fluorescence images of immune markers (CD45 or F4/80) using GIMP 2.10.38. The merged images were overlaid with a square grid generated via the Grid plugin in ImageJ, and immune cells were manually counted within each square. Grid squares were classified as either senescence‐positive or ‐negative based on the presence or absence of SA‐β‐gal‐positive tubular cells. Immune cell density (cells per square) was then compared between senescence‐positive and ‐negative regions.

Colocalization analysis of *Cdkn2a* (p16) tubular cells with CD45‐positive leukocytes or F4/80‐positive macrophages was performed using QuPath software (Bankhead et al. 2017). The analysis strategy was based on previously established QuPath‐based region‐focused image analyses for localizing immune infiltrates relative to defined tissue structures (Apaolaza et al. 2021). A pixel classifier was trained to differentiate *Cdkn2a* (p16) signal from background across the analyzed tissue section. The identified *Cdkn2a* (p16) ‐positive signal was converted into objects using the “Create Objects” function, applying a minimum object area threshold of 10 μm^2^ to exclude small background artifacts. Manual exclusion of *Cdkn2a* (p16) ‐positive interstitial cells ensured that the analysis focused exclusively on tubular *Cdkn2a* (p16) ‐positive objects. To assess immune cell localization relative to these structures, tubular *Cdkn2a* (p16) ‐positive objects were expanded by 10 μm to generate a distance‐defined *Cdkn2a* (p16) ‐proximal region. For the purpose of this analysis, these expanded zones were considered “senescence‐proximal,” i.e., “senescent” areas, whereas all remaining tissue outside these zones within the same section was considered “non‐senescent” areas. CD45‐positive leukocytes and F4/80‐positive macrophages were independently detected using a QuPath object classifier and classified according to whether they were located within the *Cdkn2a* (p16) ‐proximal region (“senescent”) or in the remaining tissue outside these zones (“non‐senescent”). This region‐based proximity approach enabled quantification of immune cell enrichment near *Cdkn2a* (p16) ‐positive tubular structures across the analyzed tissue section and was applied in both aging and senolytic experimental settings.

### 2.7 Gene Expression Analysis (NanoString)

For NanoString gene expression analysis, RNA was isolated from FFPE kidney cortex using the QIAGEN RNeasy FFPE Kit according to the manufacturer's instructions. Because RNA was extracted from bulk cortical tissue, the resulting expression profiles reflect the combined signal from all cell types present in the kidney cortex. Gene expression profiling was performed using the NanoString Fibrosis (XT‐CSO‐MFIB2‐12) and Inflammation panels (XT‐CSO‐MIN2‐12), which provide broad coverage of SASP‐related, immune‐related, and fibrosis‐associated genes. This approach was designed to assess tissue‐level associations between senescence burden and inflammatory gene expression, using the combined Fibrosis and Inflammation panels to capture coordinated tissue responses, rather than to define cell type–specific expression patterns. Raw data were processed in nSolver Analysis Software (version 4.0). Background thresholding was applied using the mean of the negative control counts. Data normalization was performed using positive control normalization and standard codeset content normalization. Data from the two panels were merged using the MultiRLF merge function, and in cases of overlapping targets, values from the Fibrosis panel were retained.

The merged dataset was further analyzed in RStudio (version 4.3.2) using the NanoTube package (version 1.8). This analysis was designed to assess tissue‐level associations between senescence burden and inflammatory gene expression. Differential gene expression analysis was performed using limma in a linear model framework, with histological and immunohistochemical readouts used as continuous variables, including *Cdkn2a* (p16) RNA‐ISH, CD45 immunostaining, Kim1 immunostaining, and Picrosirius Red staining. Normalized log2 expression values (after NanoString background correction and normalization) were scaled gene‐wise to calculate z‐scores, and heatmaps were generated using ComplexHeatmap (version 2.18.0). For the heatmap generation, genes significantly associated with *Cdkn2a* (p16) levels across all samples were selected. Old samples were classified as *Cdkn2a* (p16) high or *Cdkn2a* (p16) low based on whether the quantified *Cdkn2a* (p16) RNAscope signal was above or below the mean value across the old cohort.

Gene set enrichment analysis was performed using the R package clusterProfiler (version 4.17.0) and Gene Ontology Biological Process terms from the mouse annotation database org.Mm.eg.db. All endogenous genes represented on the combined NanoString Fibrosis and Inflammation panels were ranked according to their limma regression coefficient for association with the quantified *Cdkn2a* (p16) RNA‐ISH signal across all samples. Enrichment was assessed using the gseGO function, and pathways with adjusted *p*‐values below 0.05 were considered significant (G. C. Yu et al. 2012).

### 2.8 Visium Spatial Transcriptomics

For Visium spatial transcriptomic analysis, we combined data from publicly available sources (Chen et al. 2025) with data from our own pilot study supported by the MHH Research Core Unit GENSEC (Genomics and Single‐Cell Core Unit).

For Visium spatial transcriptomics done at our institution, FFPE kidney tissue was arranged as multi‐block arrays and processed on Visium Spatial Gene Expression slides according to the manufacturer's instructions (10  Genomics, Pleasanton, USA). Library preparation was performed using the Visium Spatial Gene Expression Reagent Kits for FFPE (10  Genomics). Library quality was assessed using the Bioanalyzer High Sensitivity DNA Assay (5067–4626; Agilent Technologies, Santa Clara, USA), and concentrations were determined using the Qubit dsDNA HS Assay Kit (Q32854; Thermo Fisher Scientific, Waltham, United States). Individually barcoded libraries were pooled, denatured, and diluted according to standard Illumina protocols, and sequenced on a NovaSeq X‐Plus platform (Illumina, San Diego, United States) using a 10B reagent kit (100 cycles). Sequencing was performed with the following read configuration: 28 bp (read 1), 90 bp (read 2), and 10 bp for each index read. Sequencing yielded a median of 730 unique molecular identifier (UMI) counts per spot and 571 genes per spot, with 15,072 genes detected and a mean sequencing saturation of 99%. Data were processed using the Space Ranger pipeline (v3.1.1, 10  Genomics) with default parameters. Briefly, BCL files were demultiplexed using spaceranger mkfastq, and reads were aligned to the mm10 reference genome (refdata‐gex‐mm10‐2020‐A) using the Visium mouse transcriptome probe set (10  Genomics). UMI count matrices were generated and imported into RStudio for downstream analysis. The analysis was performed in R (v4.5.1) using RStudio Server (2025.05.1) and the Seurat package (v5.3.1). After loading the spaceranger output, we combined the data into one object. For quality control, we removed spots with less than 750 UMIs.

To improve clustering, we then combined our data with the Visium data from (Chen et al. 2025). The data (processed R objects) were downloaded from the Gene Expression Omnibus (GEO) under the accession GSE252772. We only selected the male samples at ages 12 and 92 weeks that are the closest match to our samples. To normalize the data, we used the standard log‐normalization with scaling and 5000 variable features. After running the PCA, we integrated the data with the CCAIntegration from Seurat to minimize batch effects. We selected 30 dimensions to construct the nearest‐neighbor graph and to create the UMAP. Clustering was performed using the Leiden algorithm at default resolution. Curated cell type specific genes were used in the dot plots. Another two dot plots were created with genes associated with senescence (Lu et al. 2021; Saul et al. 2022). The R packages ggplot (v3.5.2), patchwork (v1.3.1), and scCustomize (v3.0.1) were used to make the figures.

### 2.9 Statistical Analysis

Statistical analysis was performed using GraphPad Prism (version 7.0.2) and RStudio (version 4.3.2). For most datasets, comparisons between two groups were assessed using unpaired two‐tailed Student's *t*‐tests. For spatial colocalization analyses (e.g., proximal vs. distal immune cell counts), paired two‐tailed *t*‐tests were used. In all cases, *p* < 0.05 was considered statistically significant. For transcriptomic data from Nanostring analyzed in R, adjusted *p*‐values were calculated with the Benjamini‐Hochberg (BH) procedure to correct for multiple testing, and results with *p* (adjusted) < 0.05 were considered statistically significant.

## 3 Results

### 3.1 Differential *Cdkn2a* (p16) Expression in Aged Kidney Is Associated With Differences in the Inflammatory Transcriptome

**FIGURE 1:**
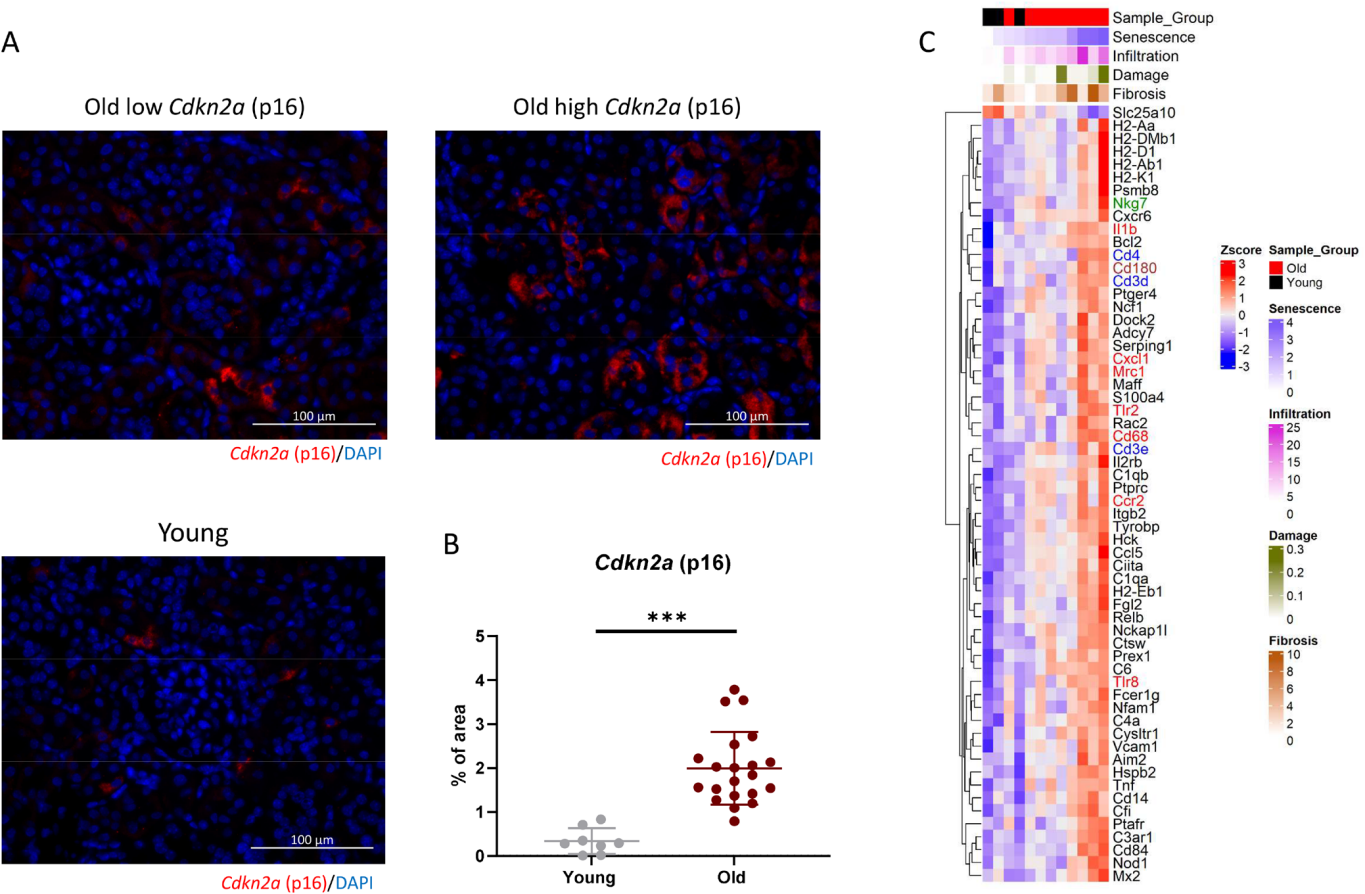
Expression of genes that change along with *Cdkn2a* (p16) levels in young and old kidneys. (A, B) RNAscope in situ hybridization for *Cdkn2a* (p16) in kidney tissue from young (3–4 months, *N* = 9) and old (22–24 months, *N* = 20) mice. Aged kidneys showed elevated but heterogeneous *Cdkn2a* (p16) expression levels. (C) Heatmap of the NanoString data from young (*N* = 3) and old (*N* = 9) mice. 60 genes are shown that changed significantly along with *Cdkn2a* (p16) RNAscope levels. The annotation includes *Cdkn2a* (p16) (senescence), CD45 (infiltration), Kim1 positive area (damage), and PSR positive area (fibrosis). Genes specific for immune cell populations were marked: Macrophages (red), T cells (blue), NK cells (green), and B cells (brown).

To assess the relationship between chronological age, biological aging, and cellular senescence, we examined *Cdkn2a* (p16) expression in kidney tissue from young and old mice using RNA‐ISH. *Cdkn2a* (p16) levels were significantly higher in kidney samples of aged mice compared to young controls. However, old mice exhibit substantial interindividual variability in *Cdkn2a* (p16) expression (Figure 1A,B).

This heterogeneity suggests that the accumulation of senescent cells during aging is not uniform, even within the same chronological age group. To further explore these differences, we performed targeted gene expression profiling focusing on SASP‐related genes. Based on *Cdkn2a* (p16) expression levels, samples from old mice were categorized into two subgroups: *Cdkn2a* (p16) low and *Cdkn2a* (p16) high.

To find out which genes are connected to this shift, we applied linear modeling using data coming from young and old kidney samples. We used expression levels of *Cdkn2a* (p16) (senescence marker), the number of infiltrating leukocytes, the amount of Kim1 expression (from injured tubular cells; Figure S2A) or Picrosirius red staining (indicating fibrosis; Figure S2B) and performed differential gene expression analysis with the data from young and old kidney samples as continuous variables to explore their association with transcriptional changes (Figure 1C). Among these, *Cdkn2a* (p16) shows the strongest association with differential gene expression, identifying 60 significantly regulated genes (adjusted *p* < 0.05). This separation indicates that *Cdkn2a* (p16) is associated with a shift in the inflammatory and fibrotic profiles in aged kidneys, independent of chronological age. Many of the genes associated with high *Cdkn2a* (p16) expression are involved in immune‐related pathways, including cytokine signaling, chemotaxis, and antigen presentation, suggesting a close association between cellular senescence and inflammation during renal aging in the cortex. Gene Ontology gene set enrichment analysis showed that genes positively associated with *Cdkn2a* (p16) were enriched for immune‐related processes, including inflammatory responses, innate and adaptive immunity, leukocyte‐mediated immunity, and leukocyte activation (Figure S2C). These findings support an association between higher *Cdkn2a* (p16) burden and immune activation during renal aging.

### 3.2 Mapping of Immune Populations

**FIGURE 2:**
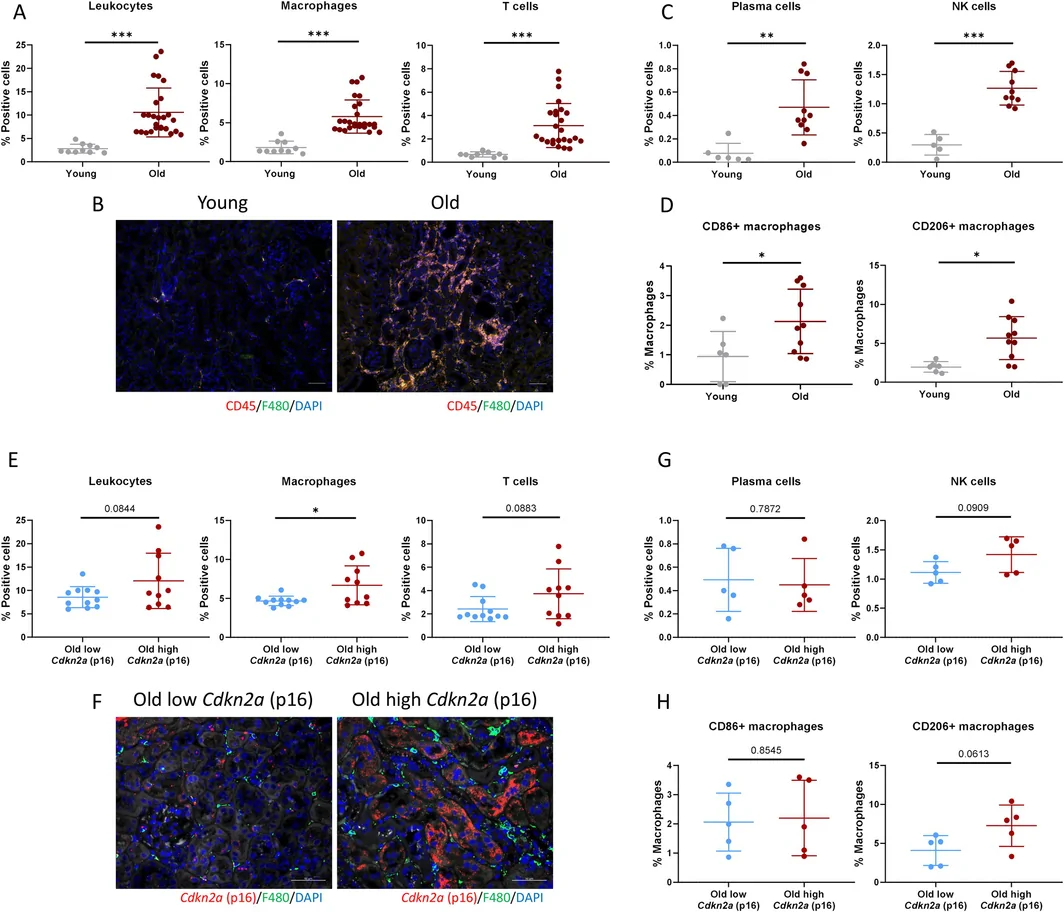
Quantification of immune cell populations in relation to aging and senescence burden. (A) Quantification of CD45‐positive leukocytes and two major immune cell sub‐populations, F4/80‐positive macrophages, and CD3‐positive T cells, showing increased abundance with age in mouse kidneys (young *N* = 10, old *N* = 26). (B) Representative immunofluorescence images of co‐stainings of CD45‐positive leukocytes (red), F4/80‐positive macrophages (green) and DAPI nuclear counterstain (blue) in young and old kidneys. (C) Quantification of CD138‐positive plasma cells and NKp46‐positive NK cells showing increased abundance in aged kidneys (young *N* = 6, old *N* = 10). (D) Quantification of CD86‐positive and CD206‐positive macrophages, showing increased abundance of both macrophage subsets in aged kidneys (young *N* = 6, old *N* = 10). (E) Quantification of CD45‐positive leukocytes, F4/80‐positive macrophages, and CD3‐positive T cells in aged kidneys stratified by low (*N* = 11) or high (*N* = 10) *Cdkn2a* (p16) expression. Macrophages were significantly increased in *Cdkn2a* (p16)‐high kidneys, whereas leukocytes and T cells showed no significant increase. (F) Representative immunofluorescence images of co‐stainings of *Cdkn2a* (p16) (red), F4/80‐positive macrophages (green) and DAPI nuclear counterstain (blue) in old *Cdkn2a* (p16)‐low and *Cdkn2a* (p16)‐high kidneys. (G) Quantification of CD138‐positive plasma cells and NKp46‐positive NK cells in aged *Cdkn2a* (p16)‐low (*N* = 5) and *Cdkn2a* (p16)‐high (*N* = 5) kidneys. No significant differences were detected. (H) Quantification of CD86‐positive and CD206‐positive macrophages in aged *Cdkn2a* (p16)‐low (*N* = 5) and *Cdkn2a* (p16)‐high (*N* = 5) kidneys. No significant differences were detected.

The analysis of the fibrosis and immune gene panels revealed immune cell‐related genes that seem to change along with *Cdkn2a* (p16) levels. To confirm that increasing age goes along with an increase in immune cell infiltration, we performed immunostainings of major immune cell populations and found a significant increase in leukocytes (CD45), macrophages (F4/80), and T cells (CD3) in old kidneys compared to young ones (Figure 2A,B). A significant increase with age was also observed in immune cell populations with rarer occurrence such as plasma cells (CD138) and NK cells (NKp46) (Figure 2C). When examining common macrophage markers of activation (pro‐inflammatory CD86‐positive macrophages, pro‐fibrotic CD206‐positive macrophages), we observed an age‐associated increase of macrophages with activated phenotypes, although the overall numbers remained small (Figure 2D).

To examine whether immune cell abundance within aged kidneys varied with senescence burden, old samples were again stratified into *Cdkn2a* (p16)‐low and *Cdkn2a* (p16)‐high groups. In this comparison, macrophages showed the strongest association with senescence burden and were more abundant in *Cdkn2a* (p16)‐high kidneys, unlike T cells (Figure 2E,F). A similar pattern was observed in kidneys from mice with *Cdkn2a* (p16) ablation, which showed reduced leukocyte and macrophage abundance (Figure S3). Plasma cells did not differ between the two groups, whereas NK cells and CD206‐positive macrophages showed only nonsignificant trends toward higher numbers in *Cdkn2a* (p16)‐high kidneys (Figure 2G,H).

### 3.3 Immune Cells Sit Preferentially in Proximity to Senescent Cells

**FIGURE 3:**
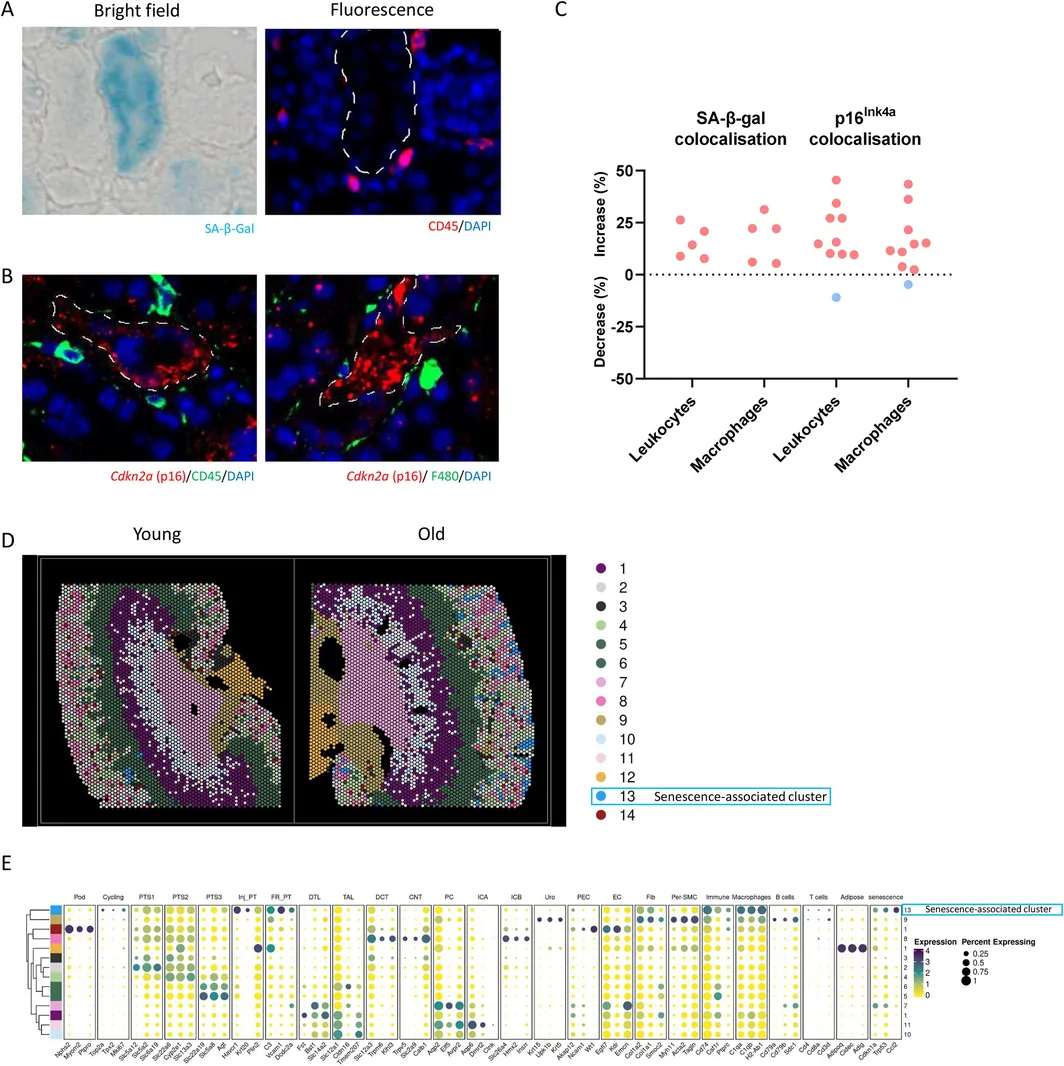
Spatial association between senescent cells and immune cells in the aging kidney. (A) Senescence‐associated β‐galactosidase (SA‐β‐gal; blue, brightfield) combined with CD45‐positive leukocytes (red, immunofluorescence) showing spatial proximity of immune cells to senescent tubules. (B) RNA in situ hybridization (RNA‐ISH) for *Cdkn2a* (p16) (red) combined with immunostaining for CD45 (green, leukocytes) or F4/80 (green, macrophages), illustrating spatial enrichment of immune cells in proximity to *Cdkn2a* (p16)‐positive tubular structures. (C) Change (in percent) in leukocyte and macrophage abundance in senescent compared with non‐senescent areas within the same tissue section. Positive values indicate higher immune cell abundance in senescent areas, whereas negative values indicate lower abundance. Data are shown for the SA‐β‐gal‐based (CD45, *N* = 5; F4/80, *N* = 5) and *Cdkn2a* (p16)‐based analysis (CD45, *N* = 10; F4/80, *N* = 10). (D) Visium spatial plots from a young (left) and an aged (right) kidney section from (Chen et al. 2025) showing the spatial distribution of transcriptomic clusters across the tissue. (E) Dot plot of curated marker genes used for cluster annotation. Dot color indicates scaled expression, and dot size indicates the fraction of spots expressing each gene. Cluster 13 is the “senescence‐associated cluster” as it combines gene expression typically derived from senescent cells, macrophages, and failed‐repair proximal tubular cells. Data comes from combined our data with data from (Chen et al. 2025). CNT, connecting tubule; DCT, distal convoluted tubule; DTL, descending thin limb; EC, endothelial cells; Fib, fibroblasts; FR_PT, failed‐ proximal tubule; ICA, intercalated cells type A; ICB, intercalated cells type B; inj_PT, injury‐like proximal tubule; PC, principal cells; PEC, parietal epithelial cells; Per‐SMC, pericytes/smooth muscle cells; Pod, podocytes; PTS1/2/3, proximal tubule segments 1/2/3; TAL, thick ascending limb; Uro, urothelial cells.

To investigate the spatial interrelationship between immune cells and senescent cells, we performed colocalization analysis. We used SA‐β‐gal staining for senescent cells followed by immunostainings for the leukocyte marker CD45 and the macrophage marker F4/80 (Figure 3A,C). This analysis revealed a significant enrichment of both CD45‐ and F4/80‐positive cells within SA‐β‐gal‐positive regions, supporting a spatial association between senescent and immune cell populations (Figures 3C and S4A). Notably, CD45‐positive cells were not double‐positive but rather situated in close proximity to SA‐β‐gal stained tubules.

To further validate these findings, we visualized *Cdkn2a* (p16) RNA‐ISH using high‐resolution imaging, combined with immunostaining (Figure 3B,C). Consistent with our initial observations, immune cells, particularly macrophages, were significantly more likely to cluster around *Cdkn2a* (p16)‐positive senescent cells than in areas without a *Cdkn2a* (p16) signal (Figures 3C and S4B). Notably, *Cdkn2a* (p16) expression is primarily localized to tubular epithelial cells. Interstitial *Cdkn2a* (p16)‐positive cells were rare and, when present, were excluded from the analysis (Figure S5).

To complement the microscopy‐based colocalization analyses, we performed supplementary Visium spatial transcriptomic analysis of aged kidneys combined with publicly available data (Chen et al. 2025). This revealed a cortex‐restricted cluster, consistent with the cortical localization of *Cdkn2a* (p16)‐positive tubular structures observed by RNA‐ISH (Figures 3D,E and S6A). Based on curated marker‐gene expression, cluster 13 exhibited a senescence‐associated transcriptomic profile, including increased expression of *Cdkn1a*, *Trp53*, *Serpine1*, *Tgfb1*, and *Ccl2*, together with immune‐associated signatures and a prominent macrophage‐associated component. In addition, the cluster showed features of failed‐repair proximal tubules, including expression of *Havcr1* (encoding protein Kim1), *Vcam1*, and *C3* (Figures 3E and S6B,C).

### 3.4 Senolysis Disrupts Immune Clusters and Reshapes the Immune Microenvironment

**FIGURE 4:**
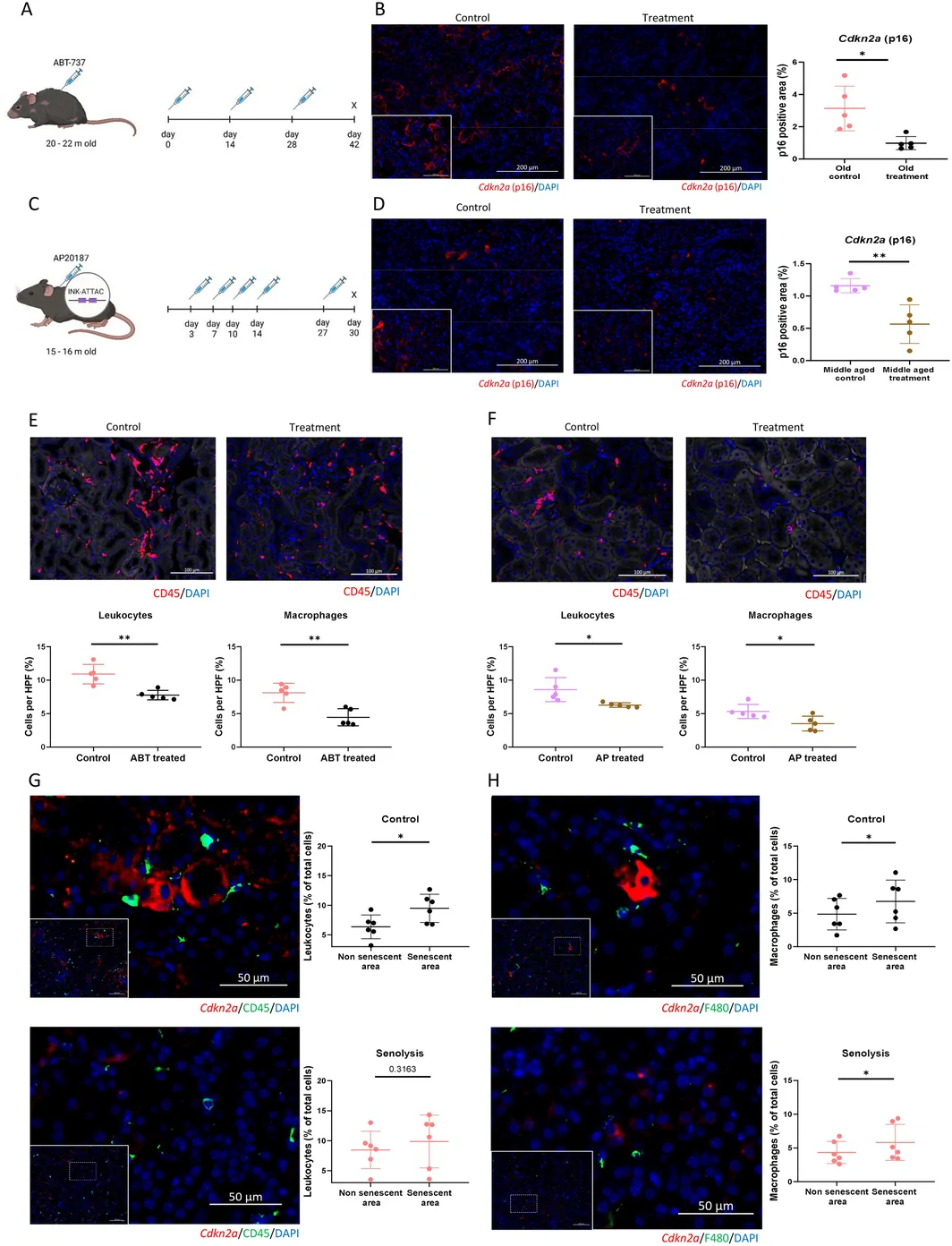
Effects of senolytic treatment on inflammaging. (A) Experimental scheme for ABT‐737‐mediated senolysis in old mice (20–22 months; *N* = 5 in each group). (B) Representative RNA‐ISH images from ABT‐737‐treated mice and respective controls (*Cdkn2a* (p16), red; DAPI, blue). Quantification revealed a significant reduction in *Cdkn2a* (p16) signal after senolysis. (C) Experimental scheme of senolysis in middle‐aged INK‐ATTAC mice. (D) Representative RNA‐ISH images from AP‐treated INK‐ATTAC mice and respective controls (*Cdkn2a* (p16), red; DAPI, blue). Quantification revealed a significant reduction in *Cdkn2a* (p16) signal after senolysis. In B and D, the larger images were acquired at 20× magnification, while the insets show representative images acquired at 40× magnification. (E) ABT‐737 treatment resulted in a significant decrease in leukocytes and macrophages compared to controls. (F) Similarly, AP administration in INK‐ATTAC mice resulted in a reduction in leukocytes and macrophages relative to controls. (G) Representative images illustrating the colocalization of CD45‐positive leukocytes with *Cdkn2a* (p16)‐positive senescent cells. Quantification revealed a significantly higher number of leukocytes that colocalized with senescent cells in vehicle‐treated kidneys. This difference was no longer detectable after senolysis. (H) Representative images illustrating the colocalization of macrophages with *Cdkn2a* (p16)‐positive senescent cells. A significantly higher number of macrophages colocalized with senescent cells. This difference remained significant, although with overall reduced numbers, after ABT‐737 treatment. For G and H, higher‐magnification views are shown, with the corresponding full microscopic field included as an inset.

To evaluate whether targeting senescent cells can modulate the immune and inflammatory landscape of the kidney, we applied two senolytic strategies in old and middle‐aged mice. We used a group of old mice (20–22 months) and treated them with ABT‐737 for 6 weeks (Figure 4A), which resulted in a significant reduction in *Cdkn2a* (p16) signal (Figure 4B). The second group consisted of middle‐aged INK‐ATTAC mice (15–16 months) (Figure 4C). Treatment with AP20187 led to a significant reduction in senescent cells in this model as well (Figure 4D).

The ABT‐737 experiments were performed in old wild‐type mice to assess senolytic effects in established kidney aging, whereas the INK‐ATTAC experiments were conducted in middle‐aged mice to examine whether reducing *Cdkn2a* (p16)‐positive cells at an earlier stage is already sufficient to decrease immune cell accumulation. Interestingly, the higher senescent cell burden in the kidneys of the old mice could be reduced to levels comparable to those of middle‐aged animals. Furthermore, the histological analysis showed a decrease in immune infiltration, both for leukocytes and macrophages, in both models (Figure 4E,F). These results indicate that immune cells were preferentially enriched in proximity to persisting senescent cells, consistent with an inflammatory microenvironment.

To investigate the exact nature of this connection, we sought to determine whether senescent cells arise in response to invading immune cells or whether senescent cells themselves attract immune cells, possibly through SASP. We performed an additional colocalization analysis in the old mice treated with either ABT‐737 or vehicle. Similar to what we had seen before, in control samples a strong clustering of immune cells was seen around senescent cells (Figure 4G,H, top panels), whereas in ABT‐737‐treated samples, this relationship disappeared and leukocytes were distributed more evenly throughout the kidney tissue (Figure 4G). Interestingly, in the case of macrophages, we observed a significant spatial correlation not only in vehicle‐treated kidneys but also in ABT‐737‐treated kidneys (Figure 4H). While the overall number of macrophages decreased, macrophages were still more frequently located near the remaining senescent islets.

## 4 Discussion

We show that in aged kidneys, senolysis not only reduced the burden of senescent cells but also diminished the local enrichment of leukocytes near remaining senescent tubular structures. Aged kidneys exhibited substantial heterogeneity in *Cdkn2a* (p16) expression, which was associated with distinct inflammatory and SASP‐related transcriptional profiles. Immune cells, particularly macrophages, were more abundant in aged compared with young, and macrophage abundance showed the strongest association with *Cdkn2a* (p16) burden. Spatial analyses using SA‐β‐gal staining, *Cdkn2a* (p16) RNA‐ISH, and complementary Visium spatial transcriptomics consistently demonstrated preferential accumulation of immune cells in proximity to senescent tubular structures. Following senolytic treatment, both senescent cell burden and immune cell infiltration were reduced in aged and middle‐aged mice. Our study provides quantitative spatial evidence that senescence‐associated inflammatory niches in the aging kidney can be modulated by senolytic intervention.

Our data indicate that immune cell accumulation in the aging kidney cortex is spatially organized rather than uniformly distributed throughout the tissue. Leukocytes were overall enriched in proximity to *Cdkn2a* (p16)‐positive and SA‐β‐gal‐positive cortical tubular regions, suggesting that senescence‐associated tubular structures form localized inflammatory niches even in the absence of overt injury. This cortical localization is consistent with earlier studies demonstrating prominent age‐associated p16^*Ink4a*^ expression in cortical tubular compartments and its association with tubular atrophy and interstitial fibrosis (Melk et al. 2005, 2004). A similar dense intracellular *Cdkn2a* (p16) RNA‐ISH pattern has been observed in renal tubular cells following kidney injury (Nath et al. 2024). Within this broader immune cell enrichment, macrophages represented one prominent component, as they not only accumulated near senescent tubules but also showed the strongest association with *Cdkn2a* (p16) burden at the whole‐cortex level. These findings are consistent with the concept of inflammaging (Lefèvre et al. 2021; Sinning et al. 2023; X. Yu et al. 2023), in which chronic low‐grade inflammatory microenvironments emerge during tissue aging and contribute to progressive tissue remodeling. The prominent macrophage accumulation observed in the present study is also consistent with our previous observations linking increased macrophage density to impaired renal outcome and progression toward end‐stage renal disease (Pfenning et al. 2023).

The supplementary Visium spatial transcriptomic analysis independently supported this interpretation by identifying a cortex‐restricted cluster enriched for senescence‐associated, inflammatory, and macrophage‐related transcriptional signatures. This cluster displayed features consistent with a failed‐repair proximal tubular state, including expression of *Havcr1*, *Vcam1*, and *C3*, together with senescence‐associated genes such as *Cdkn1a*, *Serpine1*, *Tgfb1*, and *Ccl2*. Similar inflammatory failed‐repair tubular states have previously been linked to macrophage‐rich microenvironments and persistent immune activation in injured and aging kidneys (Gerhardt et al. 2021; Kirita et al. 2020; Polonsky et al. 2024; Xuanyuan et al. 2025). The cortical localization of this senescence‐associated transcriptomic neighborhood was consistent with the predominantly cortical distribution of *Cdkn2a* (p16) RNA‐ISH signal observed in adjacent tissue regions. Together, these findings support the presence of senescence‐associated inflammatory niches in the aging kidney cortex.

Senolytic intervention altered the spatial relationship between senescent cells and immune cells in the aging kidney. In aged mice, ABT‐737 treatment reduced both *Cdkn2a* (p16) expression and overall immune cell abundance, while a similar, though less pronounced, effect was observed in the middle‐aged INK‐ATTAC model. These findings suggest that senescence‐associated inflammatory niches are at least partially reversible and may already be modifiable at earlier stages of tissue aging. Notably, macrophages remained preferentially enriched near residual senescent tubular structures after senolytic treatment. One possible explanation is that remaining senescent cells may continue to provide local chemokine signals sufficient to maintain focal immune cell accumulation (Sinning et al. 2023). In this context, the Ccl2‐Ccr2 axis is of particular interest, as senescent cells have previously been shown to recruit Ccr2‐positive myeloid cells during senescence surveillance (Lefèvre et al. 2021). Consistent with this concept, the presented combined Visium analysis identified expression of *Ccl2* within the senescence‐associated cortical cluster, while senescence‐high kidneys showed increased *Ccr2* expression in Nanostring analysis together with macrophage‐associated signatures. In addition, macrophages may remain more prominently detectable than other immune cell populations because they can persist locally within tissues and participate in the clearance of damaged or senescent cells (Elliott et al. 2009; Liu et al. 2020; Q. Zhu et al. 2022). However, the present data do not allow direct distinction between persistent recruitment, local retention, or altered immune cell turnover following senolytic treatment.

A notable finding of our study was the substantial heterogeneity in *Cdkn2a* (p16) expression among aged kidneys, despite similar chronological age and a high level of homogeneity expected among genetically identical inbred mice. Kidneys with higher *Cdkn2a* (p16) burden exhibited distinct inflammatory and SASP‐associated transcriptional profiles together with increased immune cell abundance, particularly macrophages. These observations support the concept that biological aging of the kidney is heterogeneous and that senescence burden may more closely reflect inflammatory tissue remodeling than chronological age alone. Similar variability in senescence‐associated and inflammatory signatures has previously been described in aging human kidneys and has been linked to differences in biological rather than chronological aging trajectories (Melk et al. 2004). Importantly, our transcriptomic analyses were performed on bulk cortical tissue and therefore reflect the combined contribution of multiple renal and immune cell populations. Consequently, the observed inflammatory signatures should not be interpreted as being exclusively derived from senescent tubular cells. Rather, our data suggest that kidneys with higher cortical senescence burden exhibit broader senescence‐associated inflammatory microenvironments at the tissue level.

Our study has several limitations. Macrophages can express p16^*Ink4a*^ independently of cellular senescence (Hall et al. 2016), raising the possibility that the parallel increase of *Cdkn2a* (p16) expression and macrophage abundance in the aging kidney may, in part, reflect a senescence‐independent process. However, by employing high‐resolution visualization of RNA‐ISH combined with immunostaining, and by excluding interstitial *Cdkn2a* (p16)‐positive cells from the colocalization analysis, we demonstrated that *Cdkn2a* (p16) expression was predominantly localized to tubular epithelial cells rather than CD45‐positive immune cells. Since potential hematologic off‐target effects of ABT‐737 remained an important limitation (Cippà et al. 2011), we complemented the pharmacological model with genetic approaches. Both INK‐ATTAC mice and aged p16^*Ink4a*^ knockout mice showed reduced immune cell abundance in the absence of acute pharmacological treatment, supporting an association between reduced p16^*Ink4a*^‐associated senescence burden and reduced immune infiltration. However, both ABT‐737 treatment and INK‐ATTAC‐mediated clearance act systemically and are not specific to renal tubular cells. Therefore, the observed reduction in renal inflammation cannot be attributed exclusively to the elimination of senescent tubular cells and may also reflect the clearance of other senescent populations within or outside the kidney. Local and cell type‐specific clearance models would provide approaches to distinguish renal cell‐autonomous effects from systemic effects (Farr et al. 2023; Suda et al. 2025). In addition, the present study focused predominantly on p16^*Ink4a*^‐associated senescence. Complementary models targeting other senescent populations, including p21‐high cells, may capture senescence states not represented by p16^*Ink4a*^‐based approaches (B. S. Wang et al. 2021).

While senolytic treatment reduced immune cell accumulation, the present data do not allow direct distinction between altered immune cell recruitment, local retention, recirculation, or turnover. Likewise, indirect effects mediated through changes in tissue injury or vascular function cannot be excluded. Another limitation is the relatively short observation period following senolytic treatment, which precludes conclusions regarding the long‐term durability of these effects. Transcriptomic analyses were performed on bulk cortical tissue and therefore reflect the combined contribution of multiple renal and immune cell populations. Consequently, the observed inflammatory signatures cannot be assigned to individual cell types based on the NanoString data alone. However, the integration of complementary spatial approaches consistently supported a predominantly cortical and tubular localization of the senescence‐associated inflammatory microenvironment identified in this study. Finally, differences between murine and human immune systems may limit the direct translation of our findings to human kidney aging.

In summary, our findings support a model in which senescent tubular structures contribute to the formation of localized inflammatory niches within the aging kidney cortex. These senescence‐associated microenvironments are characterized by spatial enrichment of immune cells, particularly macrophages, and are accompanied by distinct inflammatory and failed‐repair transcriptional programs. By combining pharmacological senolysis in aged mice with inducible p16^*Ink4a*^‐expressing cell ablation in middle‐aged INK‐ATTAC mice, we deliberately addressed both reversal of established renal aging and the preventive potential of senescent‐cell ablation at earlier stages. The observation that senolytic interventions reduced both senescent‐cell burden and associated immune cell accumulation suggests that these spatially organized inflammatory states are at least partially reversible. Together, these findings provide a spatial framework for understanding inflammaging in the kidney and highlight senescence‐associated inflammatory niches as a potential target for future therapeutic strategies in renal aging.

## Author Contributions

**M.J.:** conceptualizing, drafting, editing and revising the manuscript, histological analysis, visium and nanostring analysis. **M.S.:** conceptualizing, drafting the manuscript, visium, and nanostring analysis. **L.N.:** conceptualizing, visium analysis. **J.‐C.K.:** nanostring analysis. **SvV.:** conceptualizing. **J.H.B.:** conceptualizing. **R.S.:** conceptualizing and editing the manuscript. **A.M.:** conceptualizing, supervision of analyses, drafting, editing, and revising the manuscript, and funding acquisition.

## Funding

Funded by the Deutsche Forschungsgemeinschaft (DFG, German Research Foundation) ME3696/5‐1 and SFB/TRR‐298 SIIRI—Project‐ID 426335750.

## Ethics Statement

All procedures were performed in accordance with institutional and national guidelines and were approved by the Niedersächsisches Landesamt für Verbraucherschutz und Lebensmittelsicherheit (LAVES).

## Conflicts of Interest

The authors declare no conflicts of interest.

## Supporting information


**Figure S1:** Validation of *Cdkn2a* (p16) RNA‐ISH specificity using p16^*Ink4a*^ total knockout kidneys. Representative RNAscope in situ hybridization images for *Cdkn2a* (p16) (red) with DAPI (blue) in kidneys from p16^*Ink4a*^ total knockout (KO) and wild‐type (WT) mice, shown at 10× and 40× magnification.
**Figure S2:** Fibrosis and tubular injury increase with age and Gene Ontology enrichment associated with Cdkn2a (p16) expression. (A) Immunofluorescent staining for kidney injury molecule 1 (Kim1, red) shows significantly higher tubular injury in old (*N* = 19) compared to young (*N* = 10) kidneys. (B) Picrosirius Red (PSR) staining of kidney sections from young (3–4 months, *N* = 6) and old (22–24 months, *N* = 16) mice reveals significantly increased interstitial fibrosis in aged kidneys. (C) Gene set enrichment analysis of Gene Ontology Biological Process terms. All endogenous genes represented on the combined NanoString Fibrosis and Inflammation panels were ranked according to their association with the quantified Cdkn2a (p16) RNA‐ISH signal. The 25 most significantly enriched terms are shown. Bar length represents the normalized enrichment score, and color indicates the adjusted *p*‐value.
**Figure S3:** Reduced immune‐cell abundance in old p16Ink4a total knockout kidneys. Representative immunofluorescent staining for CD45 (red) with DAPI (blue) in old p16Ink4a total knockout and wild‐type kidneys. Quantification of CD45‐positive leukocytes and F4/80‐positive macrophages shows reduced immune‐cell abundance in old p16Ink4a total knockout kidneys compared with wild‐type controls.
**Figure S4:** Comparison of immune cell infiltration between senescent and non‐senescent areas. (A) Paired t‐test of colocalization of immune cells and senescent cells (*N* = 5) reveals significantly higher presence of leukocytes and macrophages in SA‐β‐gal positive areas compared to non‐senescent regions. (B) Combined RNAscope in situ hybridization for Cdkn2a (p16) and CD45 immunostaining confirms increased immune cell infiltration near senescent cells in aged kidneys (*N* = 10). Similar analysis using F4/80 staining shows spatial association between macrophages and Cdkn2a (p16)‐positive cells (*N* = 10).
**Figure S5:** Representative figure illustrating Cdkn2a (p16) expression is derived from tubular cells while macrophages rarely show expression. RNAscope for Cdkn2a (p16) (red) was combined with F4/80 immunofluorescence (green) and DAPI (blue). While p16Ink4a expression in macrophages and other immune cells has been reported in aging‐related settings, the majority of detectable Cdkn2a (p16) signal in our samples was localized to tubular structures, consistent with earlier kidney studies showing prominent age‐associated cortical p16Ink4a expression and increased p16Ink4a in kidneys with tubular atrophy/interstitial fibrosis. For the colocalization analysis, Cdkn2a (p16)‐positive signal detected in glomerular or interstitial regions was excluded, so that the analysis was restricted to tubular Cdkn2a (p16)‐positive structures.
**Figure S6:** Supplementary Visium analysis of senescence‐associated cortical neighborhoods. (A) Representative image from the Visium pilot experiment together with Cdkn2a (p16) RNA‐ISH staining from a different section of the same sample, showing corresponding cortical localization of the senescence‐associated region. (B) Dot plot of curated marker genes from the dedifferentiated senescent proximal tubule cluster described by showing overlap with our cluster 13. (C) Dot plot of the SenMayo senescence‐associated gene set across Visium clusters, showing strong enrichment in cluster 13.

## Data Availability

The data that support the findings of this study are available from the corresponding author upon reasonable request.
