# Supplementary material for: Insights Into the Interplay Between the Senescent Cells and Immune Cells
Source: Aging Cell. 2026 Sep 3;25(9):e70698. doi: 10.1111/acel.70698 (PMC13539901; doi:10.1111/acel.70698)

Supplementary Figure 1

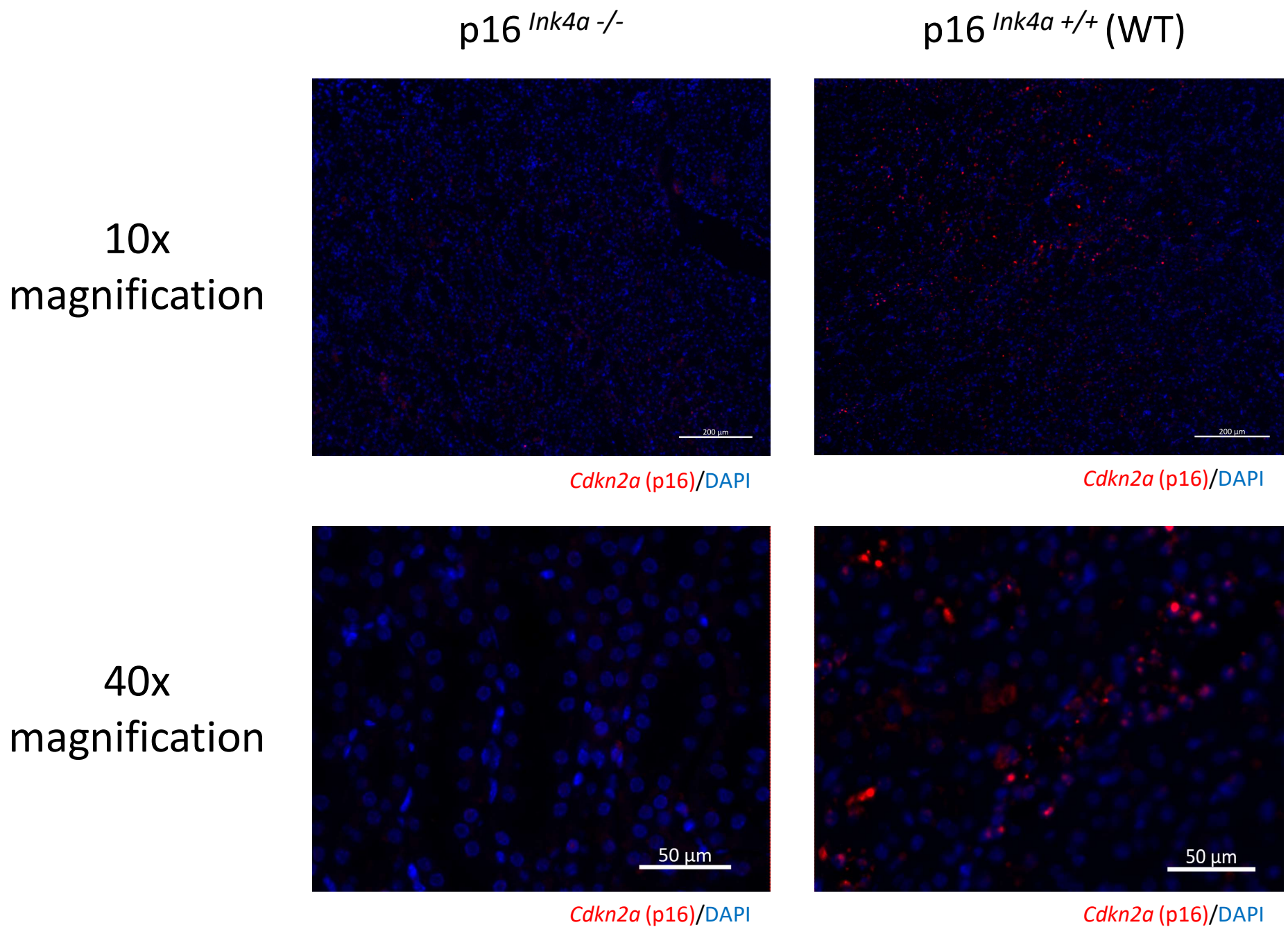

Supplementary Figure 2

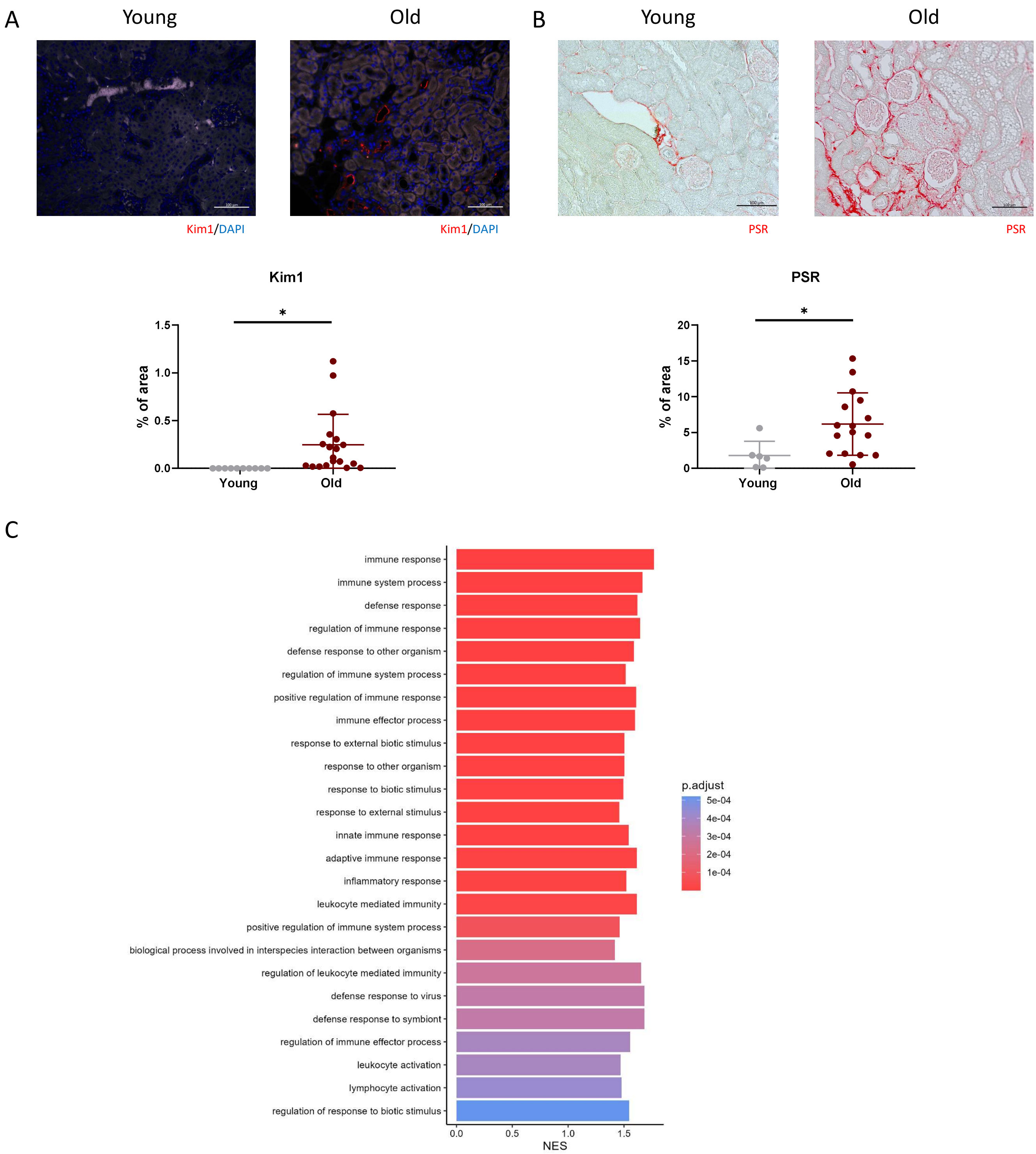

Supplementary Figure 3

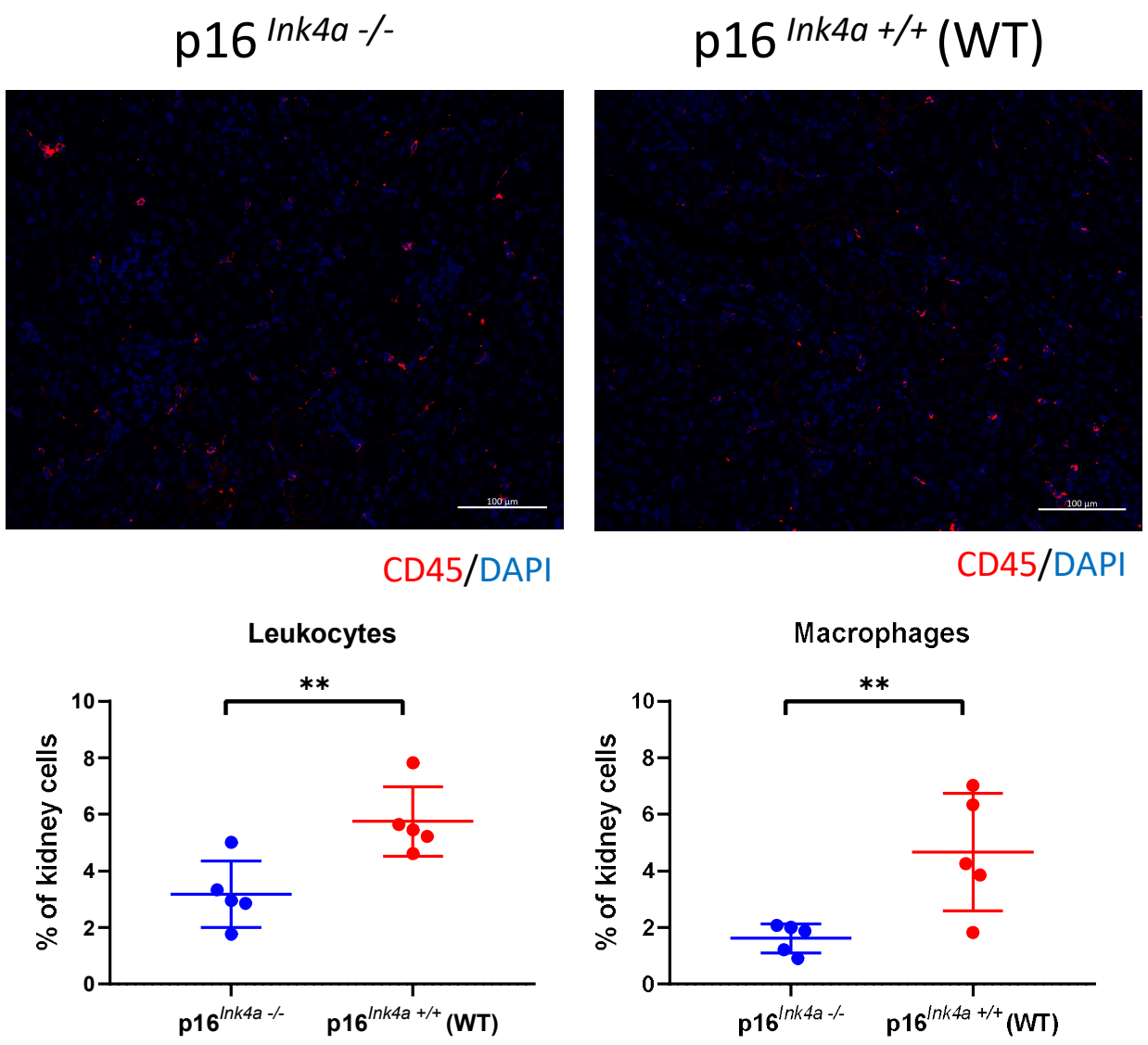

Supplementary Figure 4

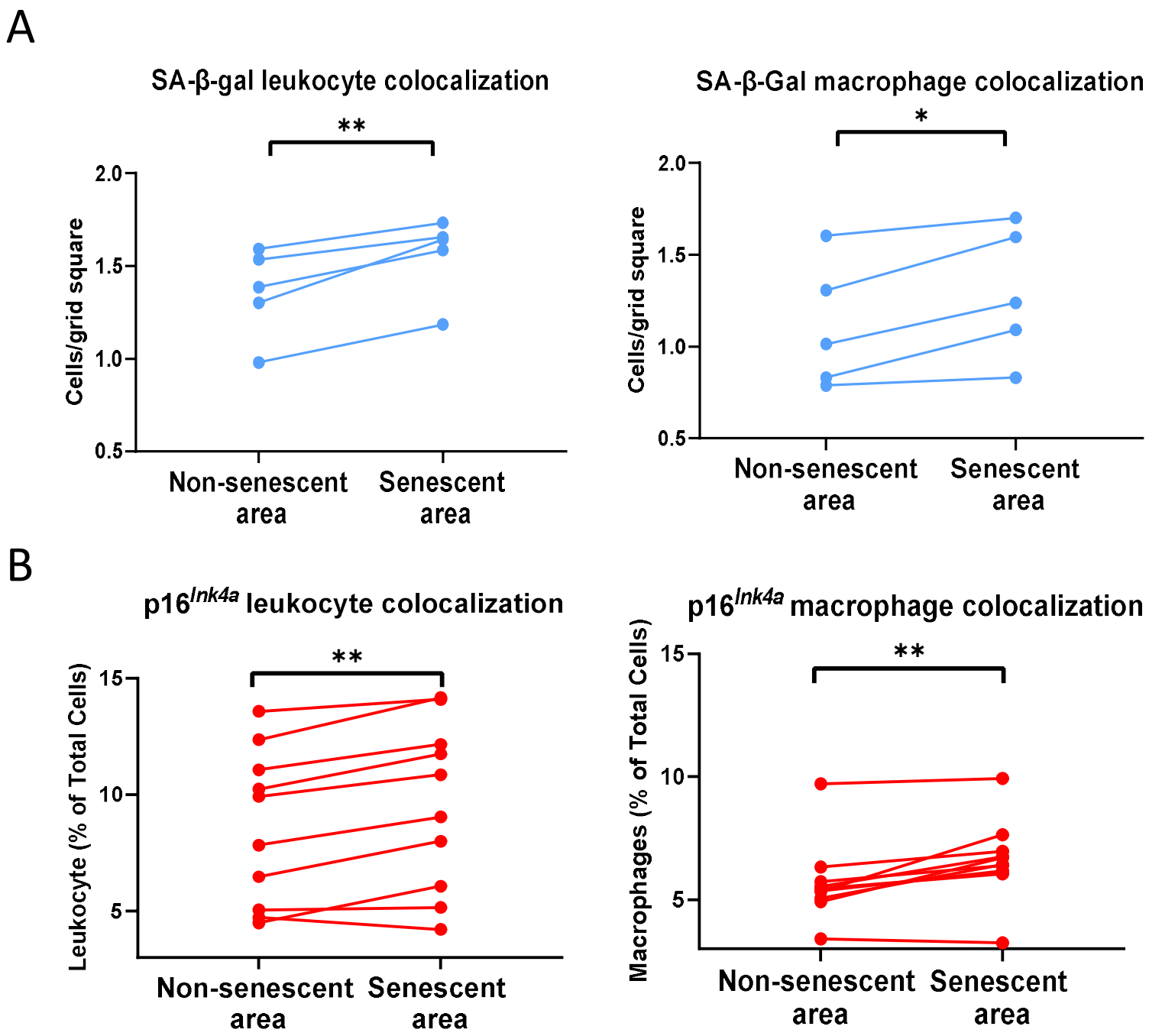

Supplementary Figure 5

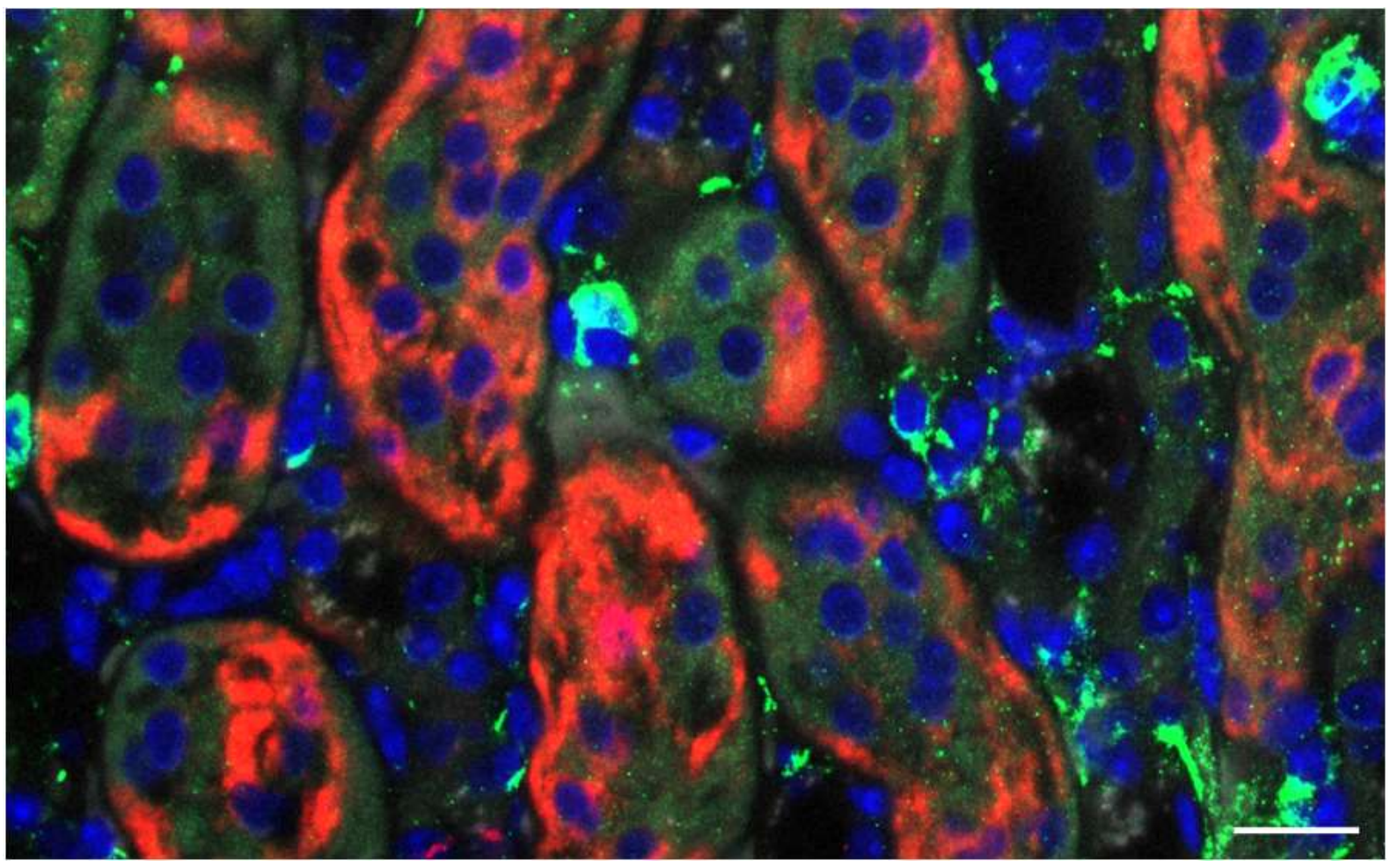

Cdkn2a (p16)/F480/DAPI

Supplementary Figure 6

A

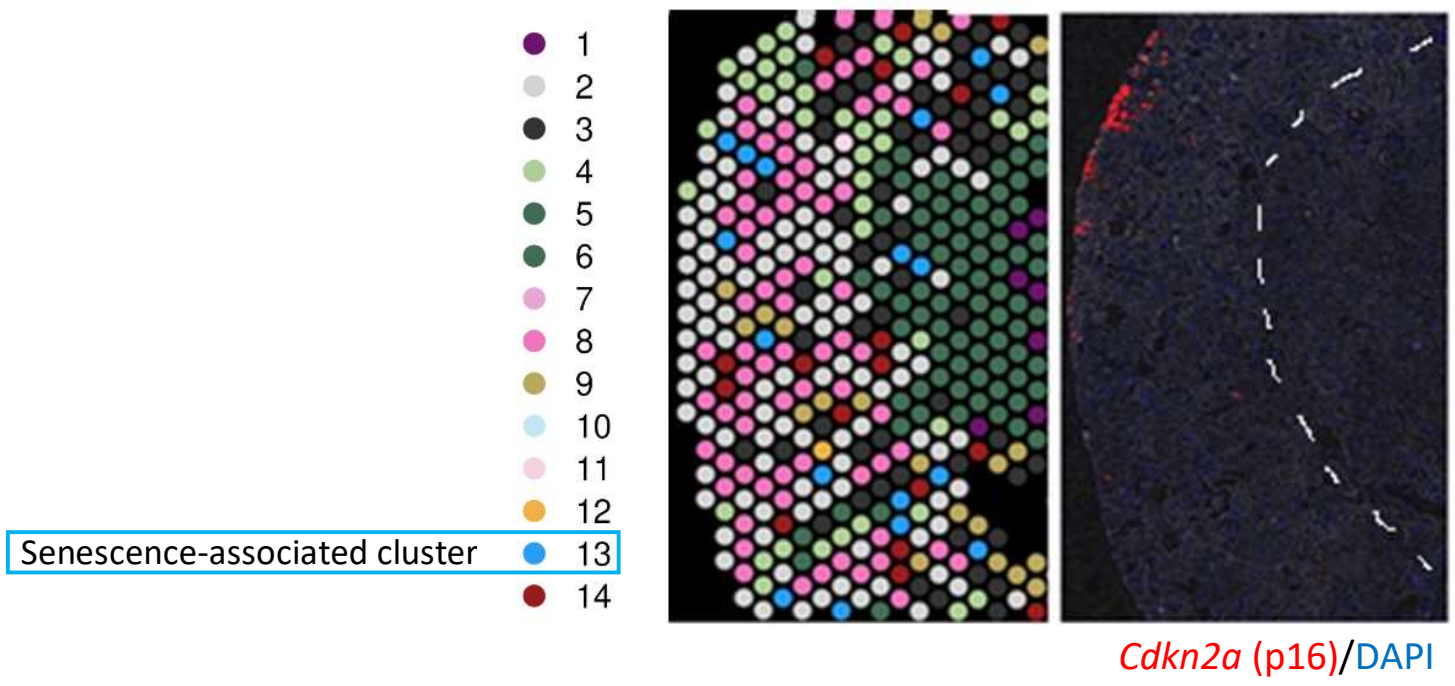

B

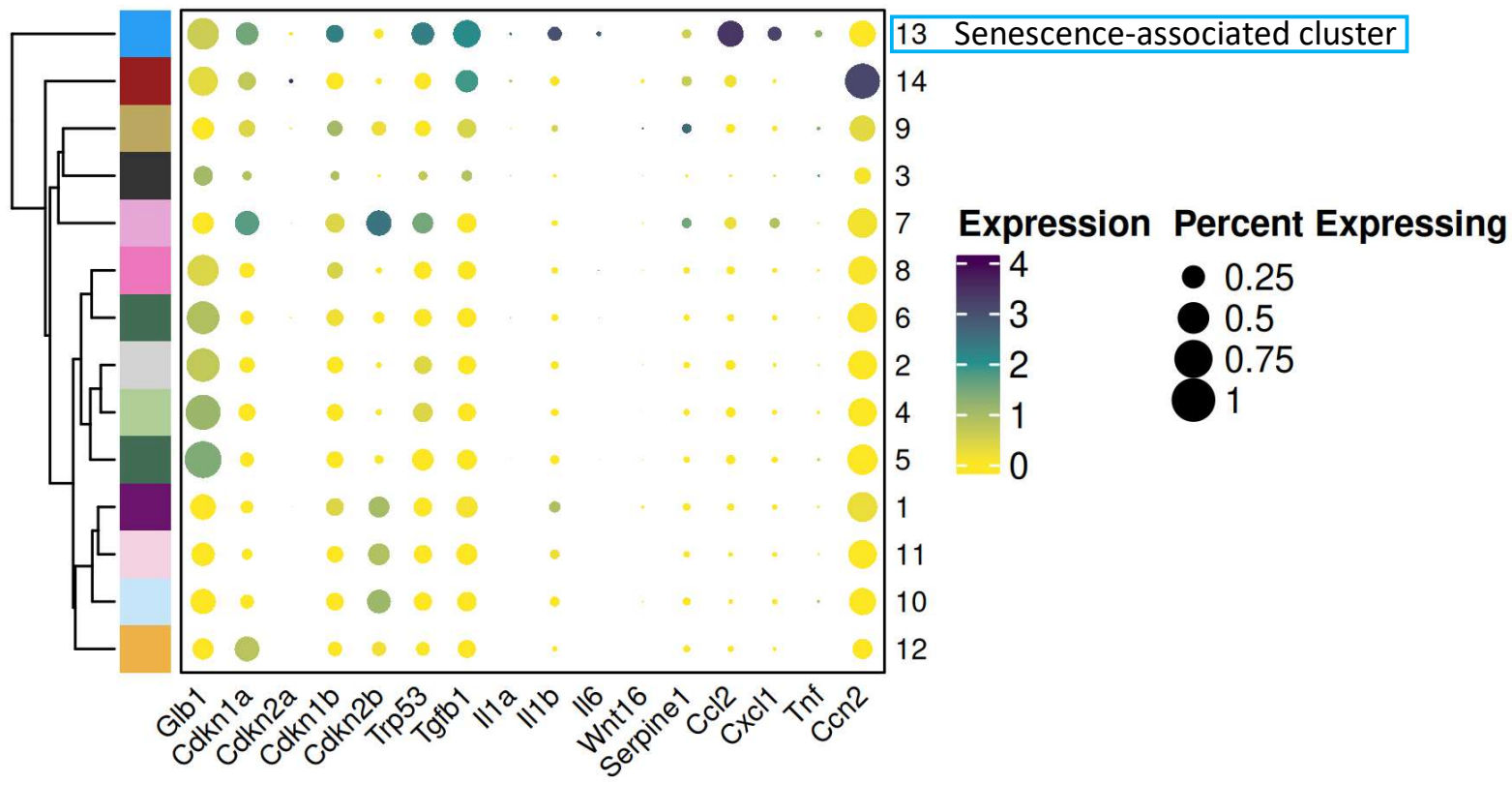

C

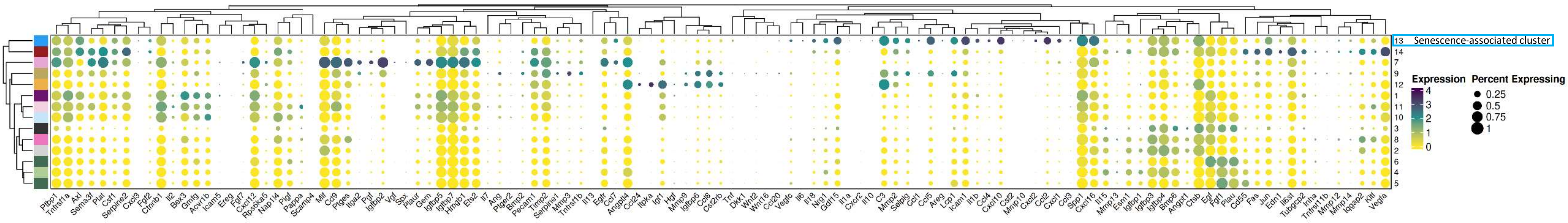

Supplement: Supplementary file 1 [file ACEL-25-e70698-s001.pdf]
